# Extrapolation of pharmacokinetics and pharmacodynamics of sunitinib in children with gastrointestinal stromal tumors

**DOI:** 10.1007/s00280-020-04221-x

**Published:** 2021-01-28

**Authors:** Reza Khosravan, Steven G. DuBois, Katherine Janeway, Erjian Wang

**Affiliations:** 1 Pfizer Inc, Oncology Clinical Pharmacology, 10646 Science Center Drive, CB10, Pfizer Oncology, La Jolla, CA 92121 USA; 2 Pediatric Hematology/Oncology, Dana-Farber/Boston Children’s Cancer and Blood Disorders Center, Harvard Medical School, Boston, MA USA

**Keywords:** Gastrointestinal stromal tumor, Sunitinib, Dose extrapolation, Pediatric cancer

## Abstract

**Purpose:**

The starting dose of sunitinib in children with gastrointestinal stromal tumors (GIST) was extrapolated based on data in adults with GIST or solid tumors and children with solid tumors.

**Methods:**

Integrated population pharmacokinetics (PK), PK/pharmacodynamics (PD), and exposure–response analyses using nonlinear mixed-effects modeling approaches were performed to extrapolate PK and PD of sunitinib in children with GIST at projected dose(s) with plasma drug exposures comparable to 50-mg/day in adults with GIST. The analysis datasets included PK/PD data in adults with GIST and adults and children with solid tumors. The effect of covariates on PK and safety/efficacy endpoints were explored.

**Results:**

Two-compartment models with lag time were successfully used to describe the PK of sunitinib and its active metabolite SU012662. PK/PD models were successfully built to describe key continuous safety and efficacy endpoints. The effect of age on sunitinib apparent clearance (CL/F) and body surface area on SU012662 CL/F was statistically significant (*P* ≤ 0.001): children who were younger or of smaller body size had lower CL/F; however, age and body size did not appear to negatively affect safety or efficacy response to plasma drug exposure.

**Conclusion:**

Based on PK, safety, and efficacy trial simulations, a sunitinib starting dose of ~ 25 mg/m^2^/day was predicted to provide comparable plasma drug exposures in children with GIST as in adults with GIST treated with 50 mg/day. However, in the absence of a tumor type effect of sunitinib on CL/F in children, the projected equivalent dose for this population would be ~ 20 mg/m^2^/day.

**Supplementary Information:**

The online version contains supplementary material available at 10.1007/s00280-020-04221-x.

## Introduction

Sunitinib is a tyrosine kinase inhibitor that targets multiple receptors in tumor proliferation and angiogenesis, including vascular endothelial growth factor receptors and platelet-derived growth factor receptors (PDGRF) [1–4]. Sunitinib is approved globally for the treatment of metastatic renal cell carcinoma (mRCC) [5, 6]. Additionally, sunitinib is approved for the treatment of gastrointestinal stromal tumors (GIST) after disease progression on imatinib and for well-differentiated pancreatic neuroendocrine tumors in patients with metastatic or unresectable, locally advanced disease [5, 6].

In 85% of adults with GIST, gain-of-function mutation in *KIT* or, more rarely, PDGRF-α (PDGFRA) proto-oncogene causes tumor transformation at a critical early stage, leading to ligand-independent activation of *KIT* [7]. Inhibition of *KIT* and PDGFRA signaling pathways by therapeutic agents (i.e., imatinib and sunitinib) has been demonstrated to have survival benefit in adult patients with metastatic GIST [8, 9]. In pediatric GIST, only 15% of tumors have detectable *KIT* or PDGFRA mutations, but tumors in pediatric GIST have a level of *KIT* expression comparable to adult GIST due to wild-type (WT) *KIT* activity [10]. Sunitinib inhibits WT *KIT* with higher potency relative to imatinib [11] and there is evidence that sunitinib may be able to slow pediatric GIST progression [12].

In adults, sunitinib 50-mg/day dosed for 4-weeks-on followed by 2-weeks-off (Schedule 4/2) has been identified as a tolerable, dose-dense regimen [13] and is the recommended starting dose/schedule for adults with GIST or mRCC [5, 6]. The safety profile is well established in adults. The most common nonhematologic adverse events (AEs) with sunitinib include fatigue/asthenia, diarrhea, mucositis/stomatitis, nausea, decreased appetite/anorexia, hypertension, vomiting, abdominal pain, and hand–foot syndrome (HFS) [5, 6]. The most common hematologic AEs include thrombocytopenia, neutropenia, anemia, and leukopenia [14].

Previous clinical experience includes studies from Janeway et al. and Agaram et al. in children with GIST and in trial ADVL0612 that included children with other solid tumors [11, 15–17]. Population-pharmacokinetic (Pop-PK) models have been previously developed to assess the PK of sunitinib and its active metabolite SU012662. These models have also been used to examine covariates that potentially contribute to variability in sunitinib and SU012662 exposure, and to predict efficacy and safety [18–21].

Sunitinib is primarily metabolized by the cytochrome P450 enzyme 3A4 (CYP3A4) to produce SU012662, which has similar biologic activity to sunitinib and constitutes 23–37% of the total exposure [5, 6]. Considering the free fraction and IC_50_ values of parent versus active metabolite, it is predicted that the majority (ie, > 80%) of the activity of the drug is driven by the parent compound; hence, the PK/PD and exposure response models were built with respect to sunitinib plasma exposures. Following multiple dosing with sunitinib 50 mg once daily doses in adult patients, the steady state concentrations of sunitinib appeared to reach or exceed the target plasma concentration of 50 ng/mL, the pre-clinically determined minimum target concentration to achieve anti-angiogenic and antitumor activity [1]. Following the administration of a single oral dose of sunitinib, the terminal half-lives of sunitinib and SU012662 40–60 h and 80–110 h, respectively, and the apparent clearance (CL/F) of sunitinib ranges from 34 to 62 L/h [5, 6].

The objectives of the current study were to develop a Pop-PK model for sunitinib and SU012662 using pooled PK data from all available studies in adults and children with GIST or solid tumors and to develop sequential PK/pharmacodynamics (PD) modeling/analysis with respect to key safety and efficacy endpoints, using PK-model post hoc predictions. Further, we aimed to extrapolate PK, safety, and efficacy of sunitinib for children with GIST using the developed models and to identify covariates that may account for the inter-individual variability in sunitinib PK/PD. To assess potential differences in treatment effect in pediatric GIST versus adult GIST, a number of on-target toxicities were evaluated to assess any impact of age on the exposure–response curve. Potential differences with respect to efficacy were evaluated by comparing the predicted objective response rate (ORR) from the model to the observed ORR from Agaram et al. [11] and Janeway et al. [15] and this also confirmed the validity of the assumptions made during model building (i.e., lack of age impact on EC_50_).

## Methods

### Study design

The PK, safety, and efficacy data collected during four studies in adults with solid tumors, the ADVL0612 study (NCT0038792) [16, 17] in children with solid tumors, and four additional studies in adults with GIST [8, 22–26] were pooled for the Pop-PK and PK/PD analyses (Online resource 1). The model-building strategy was based on modification of approaches discussed by Beal and Sheiner [27], Mandema [28], Maitre [29], and Ette [30].

A systematic multistep approach to model development was taken with base model development, random effects model development, full model development, final model development, assessment of model adequacy (goodness-of-fit), and assessment of model predictive performance (validation). For the Pop-PK and safety PK/PD analyses, all identified studies (Online resource 1) were used, whereas only studies in patients with GIST were used for efficacy PK/PD analyses.

All trials used in development of the models were conducted in accordance with the Declaration of Helsinki, International Conference on Harmonisation Good Clinical Practice guidelines, and applicable local regulatory requirements and laws. The trials were approved by the institutional review board or independent ethics committee at each center and all adult patients and children’s guardians provided written informed consent.

### Study assessments

PK, safety, and efficacy assessments were completed at pre-specified visits. The analysis of plasma PK samples was conducted by BASi, (West Lafayette, IN) for each study except Study 248-ONC-0511-002, which was conducted by Pfizer Global Research and Development Pharmacokinetics, Dynamics and Metabolism Department (Nerviano, Italy). Plasma samples were analyzed for determination of sunitinib and SU012662 using a sensitive, specific, and validated liquid chromatography–tandem mass spectrometry assay [18].

Data from safety and efficacy assessments were used for the PK/PD modeling portions. The majority of safety assessments were performed at each study visit with other safety/tumor assessments performed less frequently (e.g., once per cycle/every other cycle) following each study protocol’s requirements.

### PK model

Two previous Pop-PK analyses of sunitinib and SU01266 suggested sunitinib concentration–time data were well described using a population approach with a two-compartment PK model with first-order absorption and elimination (see Online resource 2). Thus, for this study, two separate two-compartment models with first-order absorption were used as the initial model to fit sunitinib and SU012662 concentrations [21].

The disposition kinetics were modeled using a parameterization involving apparent clearance (CL/*F*), central compartment apparent volume of distribution (Vc/*F*), apparent inter-compartmental clearance, and peripheral compartment apparent volume of distribution. A first-order absorption rate constant (*k*_a_) and a lag-time parameter (*t*_lag_) were used to characterize the absorption process. The first-order conditional estimation method with interaction estimation method was used to estimate all the parameters.

### PK/PD model

During the sequential PK/PD modeling portion, safety endpoints, consisting of the more commonly occurring AEs of sunitinib, including absolute neutrophil count (ANC), platelet count (PC), left-ventricular ejection fraction (LVEF), diastolic blood pressure (BP), lymphocyte count (LC), alanine aminotransferase (ALT), aspartate aminotransferase (AST), and hemoglobin (HG) as well as efficacy endpoint sum of longest diameter (SLD) were used. The types of base models tested were transit compartments in series with feedback loop (TCSFL) model, the indirect-response model, the indirect-response tumor model with tolerance function, and Gompertz tumor model [21, 31]. Schematics of the semi-mechanistic PK/PD model with transit compartments in series plus a rebound feedback loop and the mechanism-based PK-PD Model has been previously published (Fig. 5 in Khosravan et al. [21]).

### Categorical safety endpoints

PK/PD modeling was not used for the categorical safety endpoints HFS, fatigue, nausea, and vomiting. Instead, the relationship between the average sunitinib plasma exposures up to time of the earliest worst grade (EWG) and the incidence rate was explored using ordered categorical logistic regression models. The individual average sunitinib exposure up to time of EWG was calculated as (Eq. 1):

1 $$\left( {{\text{total}}\,{\text{dose}}\,{\text{up}}\,{\text{to}}\,{\text{EWG}}} \right)/\left( {{\text{individual}}\,{\text{post}}\,{\text{hoc}}\,{\text{CL}}/{\text{F}}\,{\text{estimate}},\,{\text{sunitinib}}} \right)/\left( {{\text{time}}\,{\text{after}}\,{\text{first}}\,{\text{dose}}\,{\text{and}}\,{\text{up}}\,{\text{to}}\,{\text{time}}\,{\text{of}}\,{\text{EWG}}\,{\text{for}}\,{\text{categorical}}\,{\text{safety}}\,{\text{endpoint}}} \right).$$

### Model evaluation and validation

Goodness-of-fit of different models to the data was evaluated using the following criteria: change in objective function, visual inspection of different diagnostic plots, precision of parameter estimates, and decreases in both inter-individual variability and residual variability. At all stages of model development (e.g., base and final), diagnostic plots were examined to assess model adequacy and possible lack of fit. Plots of observed versus predicted values, and observed versus individual predicted values were evaluated for randomness around the line of unity. Plots of conditional weighted residual versus time were evaluated for randomness around the zero line to assess whether there were any systematic deviations with respect to time which might suggest a deficiency with the structural model.

The base and final models were validated using visual predictive check techniques containing 1000 simulations, and the median and upper and lower bounds of the 95% prediction interval (PI) for PK were compared against the observed median and confidence intervals (CIs). The number of observations that was not within the 95% PI had to be < 5% of the total number of observations, and the mean prediction profile was expected to follow the observed mean profile. Additionally, the 95% CIs of the median and the lower and upper bounds of 90% PI for the simulated data were determined to ensure they included the median and the lower and upper bounds of 90% PI for the observed data, respectively [32]. Finally, bootstrapping techniques (1000 bootstrap datasets) were applied to generate the nominal 95% CIs around the point estimates, and to confirm the 95% CI generated by the base or final models based on the asymptotic standard errors generated from the NONMEM covariance step.

### PK/PD trial simulations in pediatric and adult patients with GIST

#### *Trial simulations at 15 mg/m*^*2*^

Based on the final Pop-PK and PK/PD models, trial simulations were carried out to provide predictions with respect to PK, safety, and efficacy of sunitinib in children with GIST ages 6–11 (*n* = 210) and 12–17 years (*n* = 210) in comparison to adults (*n* = 210) with GIST. In the pediatric arm, children were assigned demographics comparable to those for children with GIST and based on pediatric growth statistics. Demographics consistent with those from the dataset for adults with GIST were assigned to the adults in the comparator arm. Sunitinib doses ranged from 6.25 mg/day up to 50 mg/day on Schedule 4/2 for children, and a 50 mg/day dose on Schedule 4/2 was used for adults in the simulations. Children within each age group treated with sunitinib 15 mg/m^2^/day (i.e., ≥ 12.5 mg/m^2^ and < 17.5 mg/m^2^) were evaluated and the predicted PK and PD profiles were compared with those of adults.

#### Trial simulations of Janeway/Agaram studies

Based on the final Pop-PK and PK/PD models established for each endpoint, additional trial simulations were carried out to provide predictions with respect to the PK, safety, and efficacy of sunitinib in a typical age and gender pediatric patient population as those from studies conducted by Janeway et al. and Agaram et al. [11, 15] (Table 1). Children with GIST (*n* = 11) in these studies were administered either the sunitinib starting dose (i.e., 25–50 mg/day; ~ 25 mg/m^2^) or the maximum dose (i.e., 25–50 mg/day, ~ 30 mg/m^2^; Online resource 9) [11, 15]. Trial simulations (*n* = 200) were based on these doses. The comparator arm included adults with GIST (*n* = 11) administered 50 mg/day on Schedule 4/2.

**Table 1:** Subject baseline characteristics: continuous variables

Variable at baseline	*n*	Mean ± SD	Median (range)
Age, years	506	53 ± 16.2	55 (3–84)
Body weight, kg	500	71.8 ± 19.7	38.8 (14.8–150)
Height, cm	488	168 ± 12.8	143 (102–185)
Body surface area, m^2^	486	1.8 ± 0.287	1.26 (0.66–2.58)
AST, U/L	469	27.4 ± 18.3	24.0 (12–45)
ALT, U/L	502	25.5 ± 19.7	19.0 (9–67)
Creatinine clearance, mL/min	496	103 ± 39.8	12.0 (4–16)
Diastolic BP, mmHg	500	72.4 ± 11.2	38.8 (14.8–150)
ANC, 10^9^/L	501	5.27 ± 2.9	4.55 (1.15–21.8)
Platelet count, 10^9^/L	500	319 ± 132	290 (87–939)
Lymphocyte count, 10^9^/L	466	1.48 ± 0.86	1.34 (0.13–12.7)
Hemoglobin, g/dL	501	11.9 ± 1.67	11.8 (7.8–18.5)
LVEF, %	377	63.3 ± 7.21	63 (35.6–84)

*ALT* alanine amino transferase, *ANC* absolute neutrophil count, *AST* aspartate aminotransferase, *BP* diastolic blood pressure, *LVEF* left-ventricular ejection fraction, *SD* standard deviation

## Results

### Patient baseline characteristics and covariates

A total of 506 patients (including 35 children) in the nine studies were used for the Pop-PK and PK/PD analysis (Online resource 1). Descriptive statistics of the patient baseline characteristics and covariates considered in building the Pop-PK model for continuous and categorical variables are shown in Table 1 and Online resource 3, respectively.

### PK models

A two-compartmental model with first-order absorption and elimination rates was developed for the base models of sunitinib and SU012662. The effect of inclusion of *t*_lag_ was tested and appeared to result in a significant decrease in objective function value for both sunitinib and SU012662; hence, *t*_lag_ for absorption was included and estimated in the base model for both agents. The effects of different covariates on CL/*F*, Vc/*F*, and *k*_a_ were examined using forward selection and backward elimination procedures for both agents.

Key PK parameters in the final model with significant (*α* = 0.001) covariate effect for sunitinib and SU012662 are shown below.

For sunitinib (Eqs. 2 and 3):

2 $$\begin{aligned} {\text{CL}}/F \, & = { 5}0.{7} \times \left( {{1}{-}0.00{578} \times \left( {{\text{AGE}} - {55}} \right){-}0.000{269} \times \left( {{\text{AGE}}{-}{55}} \right)^{{2}} } \right) \times \left( {{1}{-}0.0{973} \times {\text{BEC}}_{0} } \right) \\ & \quad \times \left( {{1}{-}0.{185} \times {\text{RAC}}_{{{\text{Asian}}}} } \right) \times \left( {{1}{-}0.{169} \times {\text{SEX}}_{{\text{F}}} } \right) \times \left( {{1}{-}0.{274} \times {\text{TUM}}_{{{\text{Solid}}}} } \right), \\ \end{aligned}$$

3 $${\text{Vc}}/F \, = { 316}0 \times \left( {{\text{AGE}}/{55}} \right)^{{0.{295}}} \times \left( {{\text{BBSA}}/{1}.{81}} \right)^{{{1}.0{5}}} \times \left( {{1}{-}0.{299} \times {\text{TUM}}_{{{\text{Solid}}}} } \right),$$

wherein AGE is patient age in years, BEC_0_ is baseline Eastern Cooperative Oncology Group performance status (ECOG PS) 0, RAC is race, SEX_F_ is female, TUM_Solid_ is solid tumors, and BBSA is baseline body surface area.

Sunitinib CL/F increased and subsequently decreased with age and decreased with baseline ECOG PS 0 (− 9.73%), Asian race (− 18.5%), females (− 16.9%), and patients with solid tumors (− 27.4%). Sunitinib central Vc/F increased with age (2.95% per year for patients aged > 55 years) and BBSA and decreased in patients with solid tumors (− 29.9%).

For SU012662 (Eqs. 4 and 5):

4 $${\text{CL}}/F \, = { 22}.{1} \times \left( {{\text{BBSA}}/{1}.{81}} \right)^{{{1}.{12}}} \times \left( {{1}{-}0.{225} \times {\text{SEX}}_{{\text{F}}} } \right) \times \left( {{1}{-}0.{279} \times {\text{TUM}}_{{{\text{Solid}}}} } \right),$$

5 $${\text{Vc}}/F \, = { 317}0 \times \left( {{\text{BBSA}}/{1}.{81}} \right)^{{{2}.0{1}}} \times \left( {{1}{-}0.{278} \times {\text{TUM}}_{{{\text{Solid}}}} } \right).$$

**Fig. 1:**
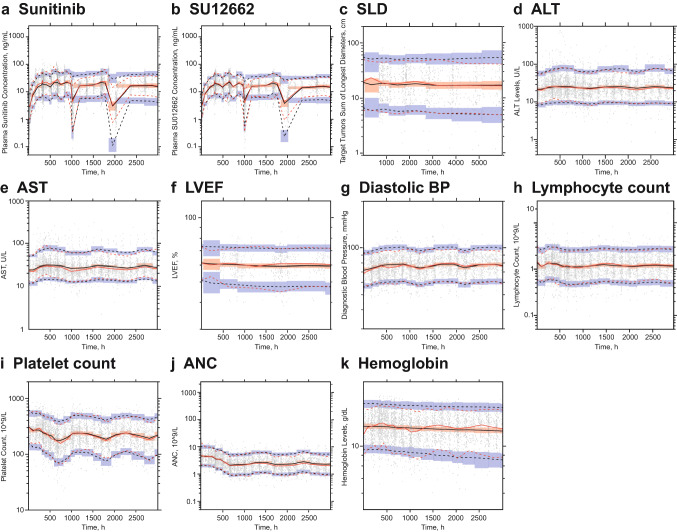
Prediction and variance-corrected visual predictive check plot (final model) for **a** plasma concentrations of sunitinib; **b** plasma concentrations of the sunitinib active metabolite SU12662; **c** efficacy endpoint sum of longest diameter in target lesions; and **d–k** selected safety endpoints. *ALT* alanine aminotransferase, *ANC* absolute neutrophil count, *AST* aspartate aminotransferase, *BP* blood pressure, *LVEF* left-ventricular ejection fraction, *SLD* sum of longest diameter

**Fig. 2:**
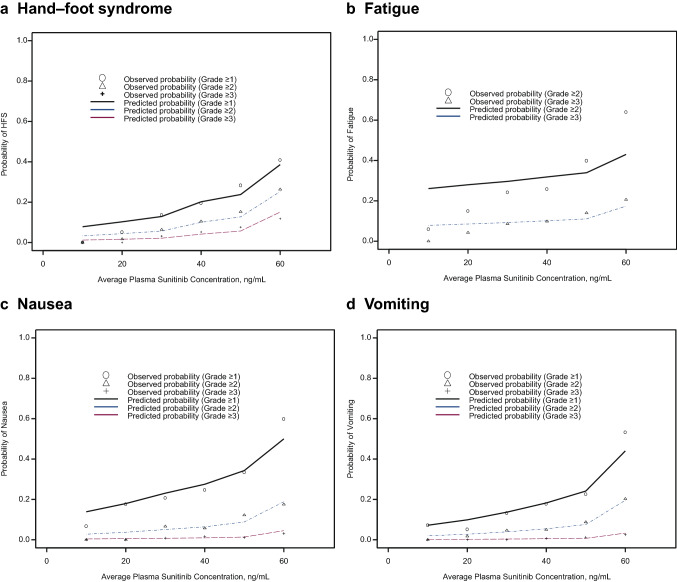
The observed and predicted probabilities of worst grades ≥ 2, and 3 for categorical safety endpoints hand–foot syndrome, fatigue, nausea, and vomiting for the final model

SU012662 CL/*F* increased with BBSA (112%) and decreased in females (− 22.5%) and in patients with solid tumors (− 27.9%). SU012662 Vc/F increased with BBSA (201%) and decreased in patients with solid tumors (− 27.8%). The goodness-of-fit of the final PK model for sunitinib and SU012662 plots was generated featuring individual and population-predicted versus observed concentrations (Figs. 1 and 2) and conditional weighted residual versus predictions or time (Online resource 4 and 5). The prediction and variance-corrected visual predictive check plot for the final models for sunitinib and SU012662 (Fig. 1) displayed good agreement between predicted and observed values. Bootstrapping techniques were applied to generate the nominal confidence intervals (CIs) around the point estimates and to confirm the 95% CI generated by the base or final models. A summary of PK parameters from the final model and bootstrapping CI values is shown in Table 2.

**Table 2:** Summary of PK parameters for sunitinib and SU012662 in the final population -PK models

Parameter	Sunitinib		SU012662	
	Final model results^a^	Bootstrap model results^b^	Final model results^a^	Bootstrap results^c^
Population mean estimates (95% CI)				
CL/*F*, L/h	50.7 (46.6–54.8)	50.6 (46.8–54.3)	22.1 (20.7–23.5)	22 (19.6–23.6)
*V*_c_/*F*, L	3160 (2952–3367)	3140 (2870–3380)	3170 (2968–3372)	3180 (2980–3390)
* k*_a_, h^−1^	0.232 (0.174–0.290)	0.246 (0.196–0.441)	0.348 (0.293–0.403)	0.37 (0.294–0.584)
* t*_lag_, h	0.527 (0.522–0.532)	0.527 (0.522–0.897)	0.519 (0.502–0.536)	0.519 (0.489–0.825)
*V*_p_/*F*, L	348 (274–422)	357 (287–475)	490 (78.4–902)	513 (298–46,000)
*Q*/*F*, L/h	1.05 (0.678–1.42)	1.05 (0.743–1.64)	0.559 (0.365–0.753)	0.598 (0.405–2.32)
Residual variability CV (95% CI)				
*σ *(*θ*_7_), %	34.3% (32.3–36.2)	33.7% (31.5–36)	31.0% (29.4–32.6)	31.0% (29.2–32.5)
Intersubject variability CV (95% CI)				
CL/*F*	36.9 (33.2–40.3)^d^	36.4 (33.2–40.3)	47.5 (42.4–52.1)^d^	47.6 (42.8–55)
*V*_c_/*F*	35.4 (29.9–40.1)^d^	35.6 (30–42.1)	48.5 (42.6–53.7)^d^	47.9 (42.7–53.7)
*k*_a_	148 (136–158)^d^	152 (136–186)	128 (109–144)^d^	134 (114–162)

*CI* confidence interval, *CL/F* apparent clearance, *CV* coefficient of variation, *k*_*a*_ absorption rate constant, *Q/F* inter-compartmental apparent clearance, *SE* standard error, *t*_*lag*_ lag time, *V*_*c*_*/F* central compartment apparent volume of distribution, *V*_*p*_*/F* peripheral compartment apparent volume of distribution

^a^95% CI was estimated as (mean–1.96 × SE–mean + 1.96 × SE)

^b^22% Run Failure (rounding error) with a single attempt; all runs were included. The numbers represent median (2.5%ile, 97.5%ile)

^c^6% Run Failure (rounding error) with a single attempt; all runs were included. The numbers represent median (2.5%ile, 97.5%ile)

^d^The shrinkage values for CL/*F*, Vc/*F* and *k*_a_ etas, the inter-individual random effects, in the base model were 5.12%,19.3% and 35.8% for sunitinib and 4.59%,11.6% and 44.6% for SU012662, respectively

### PK/PD models for efficacy and safety endpoints

#### SLD

For the efficacy endpoint SLD, a sequential indirect-response PK/PD model, with a tolerance function on *k*_a_ for loss of response (*k*_out_) and maximum effect (*E*_max_) function on zero-order constant for input (*k*_in_), was selected as the base model. Key PK/PD parameters with significant covariates in the final model are shown in Eqs. 6 and 7:

6 $${\text{BASE }} = { 17}.{4} \times \left( {{1} + 0.{3}0{6} \times {\text{BEC}}_{0} } \right) \times \left( {{1}{-}0.{4}0{3} \times {\text{SCH}}_{{{\text{CDD}}}} } \right) \times \left( {{\text{BWT}}/{71}.{2}} \right)^{{0.{513}}} ,$$

7 $$k_{{{\text{out}}}} = \, 0.000{24} \times \left( {{\text{BSLD}}/{2}0.{4}} \right)^{{{-}0.{5}0{9}}} ,$$

where BASE is baseline, SCH_CDD_ is dosing schedule with continuous daily dosing, and BSLD is baseline SLD. Mean and 95% CI values generated by the model were consistent with those generated by bootstrapping (Table 3).

**Table 3:** Summary of population parameter estimates for efficacy and safety endpoints in the final PK/PD models

Parameter	Model results^a^		Bootstrap results^b^	
	Estimate (95% CI)	Intersubject variability	Estimate (95% CI)	Intersubject variability
Efficacy endpoint				
Tumor sum of longest diameters SLD				
BASE (*θ*_1_), cm	17.4 (15.8, 19.0)	63.1 (57.2–68.4)^c^	17.5 (15.9, 19.2)	62.6 (58.1–66.7)
* k*_out_ (*θ*_2_), h^–1^	0.00024 (0.00016, 0.00032)	81.4 (70.1–91.3)^c^	0.000247 (0.000122, 0.000409)	80.1 (44.2–111)
* E*_max_ (*θ*3)	1 (FIXED)	–	1 (FIXED)	–
EC_50_ (*θ*_4_), ng/mL	162 (69.7, 254)	206 (170–237)^c^	158 (1.61, 274)	206 (161–249)
* k*_tol_ (*θ*_5_), h^–1^	0.0000447 (0.0000226, 0.0000668)	127 (97.6–150)^c^	0.0000447 (0.0000226, 0.00112)	132 (86.2–173)
*θ*BEC(BASE)	0.306 (0.133, 0.479)	–	0.3 (0.162, 0.48)	–
*θ*BWT(BASE)	0.513 (0.254, 0.772)	–	0.515 (0.198, 0.808)	–
*θ*SCH(BASE)	− 0.403 (− 0.594, − 0.212)	–	− 0.408 (− 0.525, − 0.257)	
*θ*BSLD(k_out_)	− 0.509 (− 0.799, 0.219)	–	− 0.526 (− 0.935, − 0.14)	–
Safety endpoint				
ALT				
BASE (*θ*_1_), U/L	20.5 (19.6–21.4)	44.7 (42.0–47.3)^d^	20.6 (19.6, 21.6)	44.4 (40.7–48.5)
* k*_out_ (*θ*_2_), h^–1^	0.012 (0.00757–0.0164)	162 (126–191)^d^	0.012 (0.00474, 0.0222)	163 (117–211)
* k*_PD_ (*θ*_3_), mL/ng	0.00304 (0.0026, 0.00348)	86.8 (76.8–95.8)^d^	0.00301 (0.00245, 0.00389)	87 (62.4–101)
*θ*AGE(BASE) (*θ*_5_)	− 0.188 (− 0.274, − 0.102)	–	− 0.182 (− 0.265, − 0.0874)	
AST				
BASE (*θ*_1_), U/L	24.2 (23.3, 25.1)	34.6 (33.0–36.3)^e^	24.1 (23.2, 25.1)	34.6 (30.5–39.3)
* k*_out_ (*θ*_2_), h^–1^	0.0174 (0.0174, 0.01741)	153 (131–173)^e^	0.0175 (0.014, 0.0264)	155 (131–188)
* k*_PD_ (*θ*_3_), mL/ng	0.00461 (0.00416, 0.0051)	59.5 (54.1–64.4)^e^	0.00461 (0.00423, 0.005)	59.2 (43.0–72.3)
LVEF				
BASE (θ_1_), %	61.2 (60.4, 62.0)	8.37(7.43–9.21)^f^	61.3 (60.5, 62.2)	8.28 (7.48–9.07)
* k*_out_ (*θ*_2_), h^−1^	0.00133 (0.000846, 0.00181)	93.3(20.4–130)^f^	0.0012 (0.000659, 0.00315)	92.4 (3.96–133)
* k*_PD_ (*θ*_3_), mL/ng	0.00115 (0.000876, 0.00142)	90.2 (70.1–107)^f^	0.00119 (0.000873, 0.00161)	88.7 (68.3–105)
*θ*BEC(BASE) (*θ*_5_)	0.0904 (0.0563, 0.125)	−	0.0855 (0.0684, 0.116)	−
*θ*SEX(BASE) (*θ*_6_)	0.0413 (0.0192, 0.0634)	−	0.0421 (0.0177, 0.0629)	−
Diastolic blood pressure				
BASE (*θ*_1_), mmHg	72.8 (72.0, 73.6)	10.4 (9.6–11.2)^g^	72.8 (72.0, 73.6)	10.3 (9.5–11.1)
* k*_out_ (*θ*_2_), h^–1^	0.028 (0.0271, 0.0289)	141 (102–172)^g^	0.0278 (0.0155, 0.0468)	139 (37.3–192)
* k*_PD_ (*θ*_3_), mL/ng	0.00223 (0.00203, 0.00243)	35.1 (24.2–43.3)^g^	0.00223 (0.00201, 0.00244)	34.9 (22.1–46.3)
*θ*BWT(BASE)	0.0768 (0.0376, 0.116)	–	0.076 (0.042, 0.109)	–
*θ*BBP(*k*_PD_)	− 0.0151 (− 0.0209, − 0.00926)	–	− 0.0152 (− 0.0221, − 0.00843)	–
*θ*SCH(*k*_PD_)	− 0.357 (− 0.483, − 0.231)	–	− 0.353 (− 0.458, 0.212)	–
Lymphocyte count				
BASE (*θ*_1_), 10^9^/L	1.39 (1.32, 1.46)	42.3 (39.8–44.7)^h^	1.39 (1.34, 1.46)	42 (38.9–45.4)
MTT (*θ*_2_), h	246 (234, 258)	12.8 (8.7–15.9)^h^	246 (219, 262)	12.5 (5.15–26.1)
* k*_PD_ (*θ*_3_), mL/ng	0.000771 (0.000676, 0.000866)	65.7 (55.2–74.8)^h^	0.000769 (0.00064, 0.000879)	65.9 (52.3–75.2)
POW (*θ*_4_)	0.25 (0.231, 0.269)	–	0.25 (0.176, 0.303)	–
*θ*TUM(BASE)	− 0.174 (− 0.253, − 0.0948)	–	− 0.174 (− 0.247, − 0.0893)	–
*θ*TUM(MTT)	− 0.143 (− 0.209, − 0.077)	–	− 0.146 (− 0.253, − 0.0667)	–
Platelet count				
BASE (*θ*_1_), 10^9^/L	311 (299, 323)	34.4 (31.5–37)^i^	311 (296, 325)	34.2 (32.1–36.6)
MTT (*θ*_2_), h	106 (101, 111)	24.7 (22.9–26.3)^i^	107 (90.5, 130)	23.6 (15.4–36)
* E*_*max*_ (*θ*_3_)	0.093 (0.0861, 0.0999)	37.9 (32.8–42.5)^i^	0.0962 (0.0754, 0.512)	36.1 (1.71–44.8)
EC_50_ (*θ*_4_), ng/mL	32.7 (30.2, 35.2)	31.3 (26.7–35.2)^i^	34 (27.5, 162)	30.9 (25.2–38.7)
POW (*θ*_5_)	0.0966 (0.0938, 0.0994)	–	0.0999 (0.0765, 0.147)	–
LAM (*θ*_6_)	4.77 (4.22, 5.32)	–	4.52 (1.23, 7.82)	–
*θ*TUM(BASE)	− 0.128 (− 0.191, − 0.0647)	–	− 0.127 (− 0.186, − 0.0506)	–
*θ*RAC(MTT)	− 0.152 (− 0.238, − 0.0660)	–	− 0.147 (− 0.232, − 0.0811)	–
*θ*TUM(EC_50_)	0.399 (0.245, 0.553)	–	0.386 (0.161, 0.601)	–
*θ*AGE(*E*_max_)	0.672 (0.516, 0.828)	─	0.654 (0.0551, 1.25)	–
*θ*BWT(*E*_max_)	− 0.00943 (− 0.0116, − 0.00723)	–	− 0.00928 (− 0.0123, − 0.00682)	
*θ*BEC(EC_50_)	0.217 (0.0996, 0.334)	–	0.203 (0.0771, 0.372)	–
*θ*TUM(EC_50_)	0.399 (0.245, 0.553)	–	0.386 (0.161, 0.601)	–
*θ*AGE(EC_50_)	0.571 (0.371, 0.771)	–	0.546 (− 0.108, 1.07)	–
Absolute neutrophil count				
BASE (*θ*_1_), U/L	4.71 (4.52, 4.9)	36.1 (33.3–38.6)^j^	4.72 (4.55, 4.93)	36.1 (33.3–39.1)
MTT (*θ*_2_), h	196 (189, 203)	21.5 (20–23)^j^	198 (188, 208)	20.9 (12.6–28.9)
* E*_max_ (*θ*3)	0.129 (0.120, 0.138)	17.3 (13.2–20.6)^j^	0.138 (0.116, 0.179)	16.9 (9.43–22.4)
EC_50_ (*θ*_4_), ng/mL	11.2 (9.38, 13.0)	69.6 (54.9–81.8)^j^	11.3 (7.07, 16.0)	69.3 (21.5–99.9)
POW (*θ*_5_)	0.162 (0.154, 0.170)	–	0.168 (0.144, 0.212)	–
LAM (*θ*_6_)	2.14 (1.56, 2.72)	–	1.63 (0.856, 5.06)	–
*θ*RAC(BASE)	− 0.195 (− 0.286, − 0.104)	–	− 0.188 (− 0.267, − 0.0788)	–
Hemoglobin				
BASE (*θ*_1_), g/dL	12.3 (12.2, 12.4)	12.1 (11.1–13.1)^k^	12.3 (12.2, 12.6)	12.1 (11.3–14.0)
MTT (*θ*_2_), h	1410 (1196, 1624)	96.8 (81.6–110)^k^	1234 (712, 2797)	99.5 (78.6–152)
*k*_PD_ (*θ*_3_), mL/ng	0.000277 (0.000226, 0.000328)	104 (91.0–116)^k^	0.000277 (0.000134, 0.000551)	101 (60.3–147)
POW (*θ*_4_)	0.153 (0.141, 0.165)	**–**	0.129 (0.0318, 0.181)	–
θRAC(*k*_PD_)	1.4 (0.381, 2.42)	–	0.721 (0.00104, 2.41)	–

*ALT* alanine aminotransferase, *ANC* absolute neutrophil count, *AST* aspartate aminotransferase, *BASE* baseline, *BEC* baseline Eastern Cooperative Oncology Group performance status, *BWT* baseline weight, *CI* confidence interval, *EC*_*50*_ drug concentration achieving 50% of the maximum effect, *E*_*max*_ maximum drug effect, *k*_*out*_ output elimination rate constant, *k*_*PD*_ effect first-order rate constant, *k*_*tol*_ tolerance function, *LAM* power function for the sigmoidal E_max_ model, *LVEF* left-ventricular ejection fraction, *MTT* mean transit time from the proliferation compartment to the circulation compartment, *PD* pharmacodynamic, *PK* pharmacokinetic, *POW* power function for the rebound feedback loop, *RAC* race, *SCH* dosing schedule, *SE* standard error, *SLD* sum of longest diameters, *TUM* tumor

^a^95% CI was estimated as (mean–1.96 × SE–mean + 1.96 × SE)

^b^10.5%, 13%, 19.5%, 2.5%, 4%, 3.5%, 11%, 13.5%, and 44.5% Run Failures (rounding error) with a single attempt for SLD, ALT, AST, LVEF, diastolic blood pressure, lymphocyte count, platelet count, ANC, hemoglobin, respectively; all runs were included. The numbers represent median (2.5%ile, 97.5%ile)

^c^The shrinkage values for BASE, *k*_out_, EC_50_ and *k*_tol_ etas in the base model were 0.878%, 48.3%, 35.4%, and 44.4%, respectively

^d^The shrinkage values for BASE, *k*_out_ and *k*_PD_ etas in the base model were 4.89%, 54.9% and 29.9%, respectively

^e^The shrinkage values for BASE, *k*_out_ and *k*_PD_ etas in the base model were 5.71%, 45.4% and 23.4%, respectively

^f^The shrinkage values for BASE, *k*_out_ and *k*_PD_ etas in the base model were 11.8%, 67.1% and 37.7%, respectively

^g^The shrinkage values for BASE, *k*_out_ and *k*_PD_ etas in the base model were 7.54%, 67.3% and 47.2%, respectively

^h^The shrinkage values for BASE, MTT, *k*_PD_ etas in the base model were 3.38%, 58.6% and 37.7%

^i^The shrinkage values for BASE, MTT, *E*_max_ and EC_50_ etas in the base model were 6.08%, 34.5%, 37.2% and 33.6%, respectively

^j^The shrinkage values for BASE, MTT, *E*_max_ and EC_50_ etas in the base model were 7.68%, 31.7%, 48.8% and 52.0%, respectively

^k^The shrinkage values for BASE, MTT, *k*_PD_ etas in the base model were 5.26%, 33.6% and 40.0%

EC_50_ or *k*_PD_ values are with respect to sunitinib

#### ALT, AST, LVEF, and BP

For the safety endpoint ALT, the PK/PD response model with *k*_PD_ (first-order rate constant) type effect on *k*_out_ was used as the base model, whereas the model with *k*_PD_ type effect on *k*_in_ was used as the base model for BP and LVEF.

For ALT, the key PK/PD parameter in the final model with significant (*α* = 0.001) covariate effect is shown in Eq. 8:

8 $${\text{BASE }} = { 2}0.{5} \times \left( {{\text{AGE}}/{55}} \right)^{{{-}0.{188}}} .$$

For BP, the key PK/PD parameters in the final model with significant (*α* = 0.001) covariate effect are shown in Eqs. 9 and 10:

9 $${\text{BASE }} = { 72}.{8} \times \left( {{\text{BWT}}/{71}} \right)^{{0.0{768}}}$$

10 $$k_{{{\text{PD}}}} = \, 0.00{223} \times \left( {{1}{-}0.0{151} \times \left( {{\text{BBP}}{-}{72}} \right)} \right) \times \left( {{1}{-}0.{357} \times {\text{SCH}}_{{{\text{CDD}}}} } \right),$$

wherein BBP is baseline BP and BWT is baseline body weight.

In the LVEF final model, the PK/PD parameter with significant (*α* = 0.001) covariate effect is shown in Eq. 11:

11 $${\text{BASE }} = { 61}.{2} \times \left( {{1} + 0.0{9}0{4} \times {\text{BEC}}_{0} } \right) \times \left( {{1} + 0.0{413} \times {\text{SEX}}_{{\text{F}}} } \right)$$

For AST, the PK/PD indirect-response model with *k*_PD_ first-order rate constant (i.e., slope) on *k*_out_ was selected as the base model. No significant covariates could be identified, and final and base models were determined to be the same.

#### ANC and PC

A sequential TCSFL PK/PD model with an *E*_max_ model type effect on *k*_prol_ (proliferation rate constant of the endpoint in the stem cell compartment) was used for ANC and PC.

For ANC, the PK/PD parameter BASE had a significant covariate (*α* = 0.001) effect in the final model (Eq. 12):

12 $${\text{BASE }} = { 4}.{715} \times \left( {{1}{-}0.{195} \times {\text{RAC}}_{{{\text{Asian}}}} } \right).$$

For PC, the PK/PD parameters in the final model with significant covariate effects are shown in Eqs. 13–15:

13 $${\text{MTT }} = { 1}0{6} \times \left( {{1}{-}0.{152} \times {\text{RAC}}_{{{\text{Asian}}}} } \right),$$

14 $$E_{{{\text{max}}}} = \, 0.0{93} \times \left( {{\text{AGE}}/{55}} \right)^{{0.{672}}} \times \left( {{1}{-}0.00{943} \times \left( {{\text{BWT}} - {7}0} \right)} \right),$$

15 $${\text{EC}}_{{{5}0}} = { 32}.{7} \times \left( {{1}{-}0.{217} \times {\text{BEC}}_{0} } \right) \times \, \left( {{1} + 0.{399} \times {\text{TUM}}_{{{\text{Solid}}}} } \right) \times \left( {{\text{AGE}}/{55}} \right)^{{0.{571}}} ,$$

wherein BWT is baseline weight, MTT is circulation compartment, and EC_50_ is concentration of drug producing 50% effect.

#### HG and LC

A sequential TCSFL PK/PD model with *k*_PD_ type effect on *k*_prol_ was the base model used for HG and LC.

For HG, the PK/PD parameter in the final model with significant covariate effect is shown in Eq. 16:

16 $$k_{{{\text{PD}}}} = \, 0.000{277} \times \left( {{1} + {1}.{4} \times {\text{RACE}}_{{{\text{Asian}}}} } \right).$$

For LC, the PK/PD parameters in the final model with significant covariate effect are shown in Eqs. 17 and 18:

17 $${\text{BASE }} = { 1}.{39} \times \left( {{1}{-}0.{174} \times {\text{TUM}}_{{{\text{Solid}}}} } \right)$$

18 $${\text{MTT }} = { 246} \times \left( {{1}{-}0.{143} \times {\text{TUM}}_{{{\text{Solid}}}} } \right).$$

Mean and 95% CI values generated by the efficacy and safety endpoint models were consistent with those generated by bootstrapping (Table 3).

Goodness-of-fit diagnostic plots were generated for safety endpoints AST, ALT, LVEF, ANC, BP, HB, PC, and LC and for efficacy endpoint SLD for final models with individual and population-predicted versus observed data (Online resource 4 and 5); conditional weighted residual versus predictions or time are shown in Online resource 6 and 7. Simulated predictions agreed well with observed data using visual predictive check techniques (Fig. 1).

### Categorical safety endpoints

For the categorical safety endpoints HFS, fatigue, nausea, and vomiting, the PK/PD modeling was not used; however, the relationships between the average sunitinib plasma exposures up to time of EWG and the incidence rate were explored using ordered categorical logistic regression models. Out of 502 patients included in the logistic regression analyses, there were 399, 46, 30 and 27 patients who had hand-foot syndrome with worst grades 0, 1, 2, and 3, respectively; 396, 69, 33 and 4 patients had vomiting with worst grades 0, 1, 2, and 3, respectively; 348, 111, 36 and 7 patients had nausea with worst grades 0, 1, 2, and 3, respectively; and 333, 111 and 58 patients had fatigue with worst grades 0, 2, and 3, respectively. The observed and predicted probability of highest AEs grade ≥ 1 (i.e., all grades), ≥ 2, and ≥ 3 for each categorical safety endpoint (Fig. 2) indicates concordance between observed and predicted probabilities across different grades, confirming that the logistic regression models predict the incidence of these safety events adequately. A summary of PK/PD parameters from the base and final models following bootstrapping is listed in Online resource 8; the median values from 1000 bootstrapping analysis runs were similar to the parameter estimates of the original dataset, and the bootstrapped 95% CIs overlapped with those of the models, suggesting that the final models were stable.

### Predicted PK, safety, and efficacy profiles based on final PK/PD models

Twenty trial simulations were run to generate predictions for PK, safety, and efficacy of sunitinib in children with GIST in 2 age groups, ages 6–11 and 12–17 years, and in adults with GIST in a comparator arm (see Trial simulations at 15 mg/m^2^ in methods for more details). The pooled data from all trials were assessed to generate simulated median profiles for safety endpoints ALT, ANC, AST, BP, LVEF, LC, HG, and PC, and efficacy endpoint SLD. These simulated median values, along with 95% CI, for the different endpoints are shown in Table 4. To predict the probability of incidence of HFS, nausea, vomiting, and fatigue in children with GIST, additional trial simulations based on the final PK/PD models, assuming 40% inter-subject variability, were run using the predicted mean sunitinib concentrations in each age group (Table 4). Results of the simulations indicated 47–48% lower sunitinib plasma exposures in both pediatric groups compared with adults (Table 4). Consistent with the lower drug exposures in children, there appeared to be smaller changes from baseline in the safety and efficacy measures than seen in adults.

**Table 4:** The predicted median (95% CI) for PK/safety/efficacy following multiple dosing with sunitinib 15 mg/m^2^ in children and with 50 mg in adults

PK/PD endpoint	Baseline	Median (95% CI) for each PK/PD endpoint at cycle 6 day 27/28 for each age group		
	All ages	Ages 6–11	Ages 12–17	Adults
ALT, U/L	20.5	22 (20.7, 40.3)	22.1 (20.7, 38.4)	23.7 (21, 118)
ANC, 10^9^/L	4.71	2.58 (1.6, 4.07)	2.61 (1.65, 4.17)	2.31 (1.53, 3.57)
AST, U/L	24.2	26.9 (24.8, 42.4)	27.3 (25.0, 44.7)	30.9 (25.5, 143)
BP, mmHg	72.8	76.8 (74.1, 83.6)	76.7 (74.2, 83.2)	80.6 (75.4, 95.1)
Hemoglobin, g/dL	12.3	12.0 (9.29, 12.3)	12 (9.06, 12.3)	11.7 (6.46, 12.3)
LVEF, %	61.2	59.8 (51.7, 61.0)	59.7 (52.9, 61.0)	58.6 (42.7, 60.8)
Lymphocyte count, 10^9^/L	1.39	1.29 (0.995, 1.37)	1.31 (1.06, 1.37)	1.24 (0.796, 1.36)
Platelet count, 10^9^/L	311	246 (168, 294)	235 (150, 305)	165 (47.3, 302)
SLD, % change from baseline	0.00	2.79 (–52.6, 100)	2.56 (–57.2, 99.0)	–0.685 (–65.7, 93.9)
Sunitinib trough concentration, ng/mL	0.00	20.8 (6.38, 46)	22.2 (8.11, 54.3)	44.5 (17.9, 102)
Sunitinib average concentration, ng/mL	0.00	24.7 (10.3, 51.1)	25.9 (11.7, 56.8)	48.8 (22.2, 106)
SU012662 trough concentration, ng/mL	0.00	12.0 (4.07, 31.7)	11.2 (4.19, 27.0)	23.3 (7.62, 62.2)
SU012662 average concentration, ng/mL	0.00	13.0 (5.17, 32.8)	12.0 (5.05, 27.6)	24.9 (8.85, 64.2)

*ALT* alanine amino transferase, *ANC* absolute neutrophil count, *BP* diastolic blood pressure, *CI* confidence interval, *LVEF* left-ventricular ejection fraction, *PD* pharmacodynamics, *PK* pharmacokinetics, *SLD* single longest diameter

The table shows the predicted median (95% CI) for PK/safety/efficacy on day 27/28 of cycle 6 based on the pooled data from all trial simulations following multiple dosing with sunitinib 15 mg/m^2^ in children and 50 mg in adults on Schedule 4/2 (28 days on followed by 14 days off). Baseline was set to the final model population baseline mean value for comparison of predicated relative changes of each endpoint across different age groups; median (95% CI) represents median (2.5%ile, 97.5%ile); sunitinib average concentration median (95% CI) represents mean of median (2.5%ile, 97.5%ile) values at 0, 3, 6, 9, 12, and 24 h post dose on day 27 of cycle 6

The adjusted cut-off boundaries for response and progression, based on target lesion SLD simulations, were set at 30% (with ≥ 0.5-cm increase) and 40% decrease for disease progression and partial response, respectively. The adult GIST-patient model predictions for time to progression (TTP) and objective response rate (ORR) were compared with published results [5, 33] for sunitinib (i.e., median [95% CI] Phase III TTP 26.6 [16.0–32.1] weeks and Phase III ORR 6.6% [3.8–10.5%]) to ensure they are within the range for the observed efficacy data, and to further confirm the appropriateness of the adjusted progression and response boundaries. Based on the trial simulations, the predicted medians (95% CI [i.e., 2.5%ile, 97.5%ile]) for TTP and ORR were 26.4 (24.1–29.2) weeks and 12.1% (8.2–14.9%), respectively, consistent with the observed data. Based on the analyses of the SLD PK/PD trial simulations, the predicted medians (95% CI) for TTP and ORR, respectively, were 20.9 (15.8–32.4) weeks and 4.9% (0.0–21.5%) in children aged 6–11 years and 23.8 (14.9–31.2) weeks and 8.2% (0.0–15.2%) in children aged 12–17 years, indicating slightly lower TTP and ORR in children dosed with sunitinib 15 mg/m^2^/day as compared with adults dosed at 50 mg/day. With respect to safety, all the predicted measured changes in children dosed at 15 mg/m^2^/day were less than those in adults dosed at 50 mg/day, indicating that sunitinib doses > 15 mg/m^2^/day could potentially be well-tolerated in children with GIST.

### Predicted PK, safety, and efficacy profiles in Janeway/Agaram studies and in adults with GIST, based on final PK/PD models

Trial simulations were run for children assigned demographics similar to those reported by Janeway et al*.* [15] and Agaram et al. [11] (Online resource 9; see Trial simulations of Janeway/Agaram studies in the methods for more details). Based on pooled data from all trials, the simulated median (95% CI) for safety endpoints ALT, ANC, AST, BP, HG, LVEF, LC, and PC, and efficacy endpoint SLD were generated and are shown in Online resource 10. Additional trial simulations were run to predict the probability of incidence of the AEs HFS, nausea, vomiting, and fatigue in both pediatric age groups and in adults with GIST based on the final PK/PD models. These simulation runs used predicted average sunitinib concentrations in each age group with an assumed 40% inter-subject variability. Results of the simulation runs indicated that in both the Janeway and Agaram studies, sunitinib plasma exposure was higher in children with GIST who received sunitinib at starting doses as compared with children in both age groups dosed at 15 mg/m^2^. The plasma exposure levels in the Janeway and Agaram studies, although still lower (i.e., by 12%), were much closer to those in adults with GIST receiving 50 mg/day (Table 4 and Online resource 10).

Based on the trial simulations described previously, the predicted medians (95% CI) for TTP and ORR were 24.8 (10.5–42.6) weeks and 9.0% (0.0–36.0%), respectively, in the pediatric patient population in the Janeway/Agaram studies and were 24.7 (12.7–42.6) weeks and 9.0% (0.0–27.0%) in adults with GIST. Consistent with similar drug exposures, there appeared to be similar changes from baseline in the safety and efficacy measures and, in most cases, with overlapping profiles. Based on the trial simulations, the predicted medians (95% CI) for TTP and ORR, respectively, at the maximum sunitinib dose were similar to those at the starting dose: 25.1 (10.5–42.6) weeks and 9.0% (0.0–36.0%) for patients in the Janeway and Agaram studies [11, 15] versus 24.7 (12.7–42.6) weeks and 9.0% (0.0–27.0%) in adults.

## Discussion

The quality of fit for PK, safety, and efficacy as determined for each final model were adequate to enable prediction in adults with GIST. Sunitinib 15 mg/m^2^ was the maximum tolerated dose (MTD) based on the Phase I study of children with solid tumors (Study ADVL0612; NCT00387920 [16]) and the starting dose in the Phase I study in children with GIST (Study A6181196; NCT01396148 [34]). The projection of MTD in ADVL0612 was conducted in pediatric patients who had central nervous system solid tumors and were heavily pretreated [16] and thus may have underestimated MTD in the context of pediatric patients with GIST. In study A6181196, children with GIST were started at sunitinib 15 mg/m^2^ with a majority of these patient, 5 out of 6, escalated to 22 mg/m^2^ and with a further escalation to 30 mg/m^2^ for 2 of these 5 patients [34]. In these previous studies, the predicted measured changes from baseline in safety in children dosed with sunitinib 15 mg/m^2^ were fewer than those reported in adults who received 50 mg/day, which indicated doses of sunitinib higher than 15 mg/m^2^ could potentially be well-tolerated in children with GIST.

The overall safety and tolerability conclusions as well as the patient objective response data from the Janeway and Agaram studies were used to confirm the model findings; however, these studies did not include any PK or individual patient level data or lab values that could be included the population PK/PD dataset used for model development. The observed ORR within the Janeway and Agaram studies was ~ 18% [11, 15], which is higher than the median predictions, but still within the 95% CI (0.0–36.0%) for the median, confirming the predictiveness of the SLD PK/PD model in children with GIST. The median TTP of ~ 34 weeks was within the 95% CI for TTP (10.4–42.6 weeks), further confirming the validity of the tumor response model in children with GIST and also the potential antitumor effects of sunitinib regardless of age. Sunitinib plasma exposures reported by Janeway et al. and Agaram et al. were higher in patients who received maximum doses as compared with both pediatric age groups with GIST dosed at 15 mg/m^2^, and plasma exposure was similar to those in adults with GIST receiving sunitinib 50 mg/day [11, 15].

Exposure–response analyses in patients with mRCC and GIST indicated that patients with higher average daily plasma exposure of sunitinib had higher ORR, stable disease rate, TTP, and overall survival [19]. The trial simulation results for PK, safety, and efficacy indicated that sunitinib starting doses of ~ 25 mg/m^2^ would be more appropriate in children with GIST and provide PK, safety, and efficacy results comparable to adults with GIST treated with 50 mg. However, this assumes similar tumor type effect (i.e., GIST vs*.* solid tumors) on CL/*F* in children as in adults. In the absence of tumor type effect on CL/*F* in children contrary to that observed in adults, the predicted dose will be ~ 20 mg/m^2^ (i.e., 25 mg/m^2^ × [1–0.274] = 18.2 mg/m^2^; see Eq. 2).

With respect to efficacy, efforts were made to estimate the predicted TTP and ORR using the simulated SLD profile and using a correction factor for the RECIST-defined response (i.e., > 30% decrease in SLD) and progression (i.e., > 20% increase in SLD with ≥ 0.5-cm increase) boundaries. This correction factor was used to account for the inherent residual error unaccounted for by the model parameters, which were ~ 10% of baseline and, more importantly, intra-patient dose modifications, interruption, and disease progression due to non-target lesions/new lesions. Therefore, the adjusted cut-off boundaries for response and progression, based on target lesions SLD simulations, were set at 30% increase (with ≥ 0.5-cm increase) and 40% decrease for disease progression and partial response, respectively. Using the adjusted cut-off boundary, the predicted TTP and ORR values in adults with GIST were consistent with observed values in adults with GIST, further supporting the choice of the new cut-off boundaries for response and progression.

The current analysis is limited by the lack of a study performed with a starting sunitinib dose of 25 mg/m^2^ (or 20 mg/m^2^ in the absence of tumor type effect) to provide a comparison for the predicted PK, safety, and efficacy endpoints in this study. In the absence of such a study for comparison, the available patient level data from Agaram et al. [11] and Janeway et al. [15] in patients with GIST with starting or maximum doses ranging from 25 to 50 mg were used to further confirm the performance of the safety and efficacy endpoint models where possible.

It should be noted that findings from the extrapolations based on this work have been subsequently confirmed by both the integrated Pop-PK modeling and Pop-PK/PD modeling in children [35–37]. The findings from this extrapolation are also supported by the Phase I/II trial of sunitinib in children (median age 14 years [range 13–16 years]) with advanced GIST recently reported by Verschuur et al. [34] One conclusion from that trial was the tolerable dose of sunitinib for these patients was at least 20 mg/m^2^ on Schedule 4/2 [34].

## Conclusion

The PK of sunitinib and SU012662 were successfully described using two-compartment models with lag time. Also, key safety and efficacy endpoints of sunitinib in children with GIST were successfully described using mechanism-based and semi-mechanistic PK/PD models. Overall, results of simulations indicated that sunitinib benefit–risk was mainly driven by plasma drug exposure across different age groups and did not appear to be affected by younger age or lower body size. Based on the results of the PK, safety, and efficacy trial simulations, a sunitinib starting dose of ~ 15 mg/m^2^/day appears to be inadequate in the treatment of children with GIST. The simulations in the current study predict that a sunitinib starting dose of ~ 25 mg/m^2^/day, in the presence of tumor type effect (as in patients with GIST vs. patients with solid tumors) on CL/*F*, equivalent to ~ 20 mg/m^2^/day in the absence of tumor type effect on CL/*F*, to be more appropriate in children with GIST, as this dose is predicted to provide comparable plasma drug exposures and subsequently, safety and efficacy to 50 mg/day on Schedule 4/2 in adult patients with GIST.

## Supplementary Information

Below is the link to the electronic supplementary material.

Supplementary file1 (PDF 2358 KB)

## Data Availability

Upon request, and subject to certain criteria, conditions, and exceptions (see https://www.pfizer.com/science/clinical-trials/trial-data-and-results for more information), Pfizer will provide access to individual de-identified participant data from Pfizer-sponsored global interventional clinical studies conducted for medicines, vaccines, and medical devices (1) for indications that have been approved in the US and/or EU or (2) in programs that have been terminated (i.e., development for all indications has been discontinued). Pfizer will also consider requests for the protocol, data dictionary, and statistical analysis plan. Data may be requested from Pfizer trials 24 months after study completion. The de-identified participant data will be made available to researchers whose proposals meet the research criteria and other conditions, and for which an exception does not apply, via a secure portal. To gain access, data requestors must enter into a data access agreement with Pfizer.
