# Supplementary material for: Extrapolation of pharmacokinetics and pharmacodynamics of sunitinib in children with gastrointestinal stromal tumors
Source: Cancer Chemother Pharmacol. 2021 Jan 28;87(5):621–34. doi: 10.1007/s00280-020-04221-x (PMC8026416; doi:10.1007/s00280-020-04221-x)
Supplement: Supplementary file 1 — Supplementary file1 (PDF 2358 KB) [file 280_2020_4221_MOESM1_ESM.pdf]

Extrapolation of Pharmacokinetics and Pharmacodynamics of Sunitinib for Children With Gastrointestinal Stromal Tumors

Cancer Chemotherapy and Pharmacology

Reza Khosravan, Steven G. DuBois, Katherine Janeway, and Erjian Wang

Correspondence: R. Khosraven, MD, Pfizer Inc, Reza.Khosravan@pfizer.com

Online Resource 1 Characteristics of phase 1–3 studies used for population PK and PK/PD analyses

Study number	Study design / tumor	Age category	*n*[a]	Dosing schedule: sunitinib dose
248-ONC-0511-002b	Phase I / Solid tumors	Adults	27	4/2: 25, 50, 75, or 100 mg QD or QoD
RTKC-0511-005b	Phase I / Solid tumors	Adults	41	4/2 or 2/2: 50 and 75 mg QD or QoD
RTKC-0511-016b	Phase I / Solid tumors	Adults	12	2/1: 50 mg
RTKC-0511-018 (NCT00054886) [1-3]	Phase I / Solid tumors	Adults	26	2/1: 50 mg (loading dose 50–175 mg only on Day 1 of Cycle 1)
ADVL0612 [4,5]	Phase I / Solid tumors	Children	35	4/2: 15 mg/m2 or 20 mg/m2
RTKC-0511-013 (NCT00054886) [2]	Phase I/II / GIST	Adults	86	2/1: 50 mg; 2/2: 25, 50, 75 mg 4/2: 50 mg
A6181004 [6]	Phase III / GIST	Adults	217	4/2: 50 mg
A6181045 [7]	Phase I/II / GIST	Adults	36	4/2: 25, 50, 75 mg
A6181047 [8]	Phase II / GIST	Adults	26	CDD: 37.5 mg

[a] Number of PK-evaluable subjects.

[b] Pfizer data on file.

2/1 2-weeks-on/1-week-off dosing schedule, *4/2* 4-weeks-on/2-weeks-off dosing schedule, *CDD* continuous daily dosing, *GIST* gastrointestinal stromal tumor, *PD* pharmacodynamics, *PK* pharmacokinetics, *QD* once per day dosing, *QoD* every other day dosing

References

1. Grunwald V, McKay RR, Krajewski KM, Kalanovic D, Lin X, Perkins JJ, Simantov R, Choueiri TK (2015) Depth of remission is a prognostic factor for survival in patients with metastatic renal cell carcinoma. Eur Urol 67:952-958. doi: 10.1016/j.eururo.2014.12.036
2. Grunwald V, Lin X, Kalanovic D, Simantov R (2016) Early tumour shrinkage: a tool for the detection of early clinical activity in metastatic renal cell carcinoma. Eur Urol 70:1006-1015. doi: 10.1016/j.eururo.2016.05.010
3. de Velasco G, McKay RR, Lin X, Moreira RB, Simantov R, Choueiri TK (2017) Comprehensive analysis of survival outcomes in non-clear cell renal cell carcinoma patients treated in clinical trials. Clin Genitourin Cancer 15:652-660.e651. doi: 10.1016/j.clgc.2017.03.004
4. Dubois SG, Shusterman S, Ingle AM, Ahern CH, Reid JM, Wu B, Baruchel S, Glade-Bender J, Ivy P, Grier HE et al (2011) Phase I and pharmacokinetic study of sunitinib in pediatric patients with refractory solid tumors: a children's oncology group study. Clin Cancer Res 17:5113-5122. doi: 10.1158/1078-0432.ccr-11-0237
5. DuBois SG, Shusterman S, Reid JM, Ingle AM, Ahern CH, Baruchel S, Glade-Bender J, Ivy P, Adamson PC, Blaney SM (2012) Tolerability and pharmacokinetic profile of a sunitinib powder formulation in pediatric patients with refractory solid tumors: a Children's Oncology Group study. Cancer Chemother Pharmacol 69:1021-1027. doi: 10.1007/s00280-011-1798-2
6. Demetri GD, van Oosterom AT, Garrett CR, Blackstein ME, Shah MH, Verweij J, McArthur G, Judson IR, Heinrich MC, Morgan JA et al (2006) Efficacy and safety of sunitinib in patients with advanced gastrointestinal stromal tumour after failure of imatinib: a randomised controlled trial. Lancet 368:1329-1338. doi: 10.1016/S0140-6736(06)69446-4
7. Shirao K, Nishida T, Doi T, Komatsu Y, Muro K, Li Y, Ueda E, Ohtsu A (2010) Phase I/II study of sunitinib malate in Japanese patients with gastrointestinal stromal tumo after failure of prior treatment with imatinib mesylate. Invest New Drugs 28:866-875. doi: 10.1007/s10637-009-9306-9
8. George S, Blay JY, Casali PG, Le Cesne A, Stephenson P, Deprimo SE, Harmon CS, Law CN, Morgan JA, Ray-Coquard I et al (2009) Clinical evaluation of continuous daily dosing of sunitinib malate in patients with advanced gastrointestinal stromal tumour after imatinib failure. Eur J Cancer 45:1959-1968. doi: 10.1016/j.eja.2009.02.011

Online Resource 2 Prior Knowledge/Modeling Experience

Two previous population-pharmacokinetic (Pop-PK) analyses of sunitinib and its metabolite suggest that sunitinib concentration–time data were well described using a population approach with a two-compartment PK model with first-order absorption and elimination [1, 2]. In the first Pop-PK analysis, apparent clearance (CL/F) of sunitinib was estimated to be 37.6 L/h and apparent volume of distribution for the central compartment (Vc/F) of the parent was estimated to be 2230 L for a healthy 50-year-old white male weighing 75 kg with an Eastern Cooperative Oncology Group performance status (ECOG PS) 0.

Based on a Pop-PK meta-analysis of sunitinib and SU12662 [1], a conversion of 21% of the total parent to metabolite was assumed to bring the magnitude of the parameters for SU012662 to a more physiologically relevant level. CL/F was estimated at 20.5 L/h and Vc/F was estimated to be 3260 L for a healthy 50-year-old white male weighing 75 kg with an ECOG PS 0. Inter-individual variability was estimated to be 46% for CL/F and 53% for Vc/F.

In the second Pop-PK analysis, CL/F of sunitinib was estimated to be 51.8 L/h and Vc/F of the parent was estimated to be 2030 L for a healthy male non-Asian subject weighing 77.2 kg [1, 2]. Inter-individual variability was estimated to be 38% for CL/F and 43% for Vc/F. The absorption rate constant was estimated at 0.195 h^{-1}, with inter-individual variability of 80%.

Disposition of the metabolite was also described using a two-compartment model. Based upon preclinical observations, a conversion of 21% of the total parent to metabolite was assumed to bring the magnitude of the parameters to a more physiologically relevant level. CL/F was estimated at 29.6 L/h and Vc/F was estimated to be 3080 L for a healthy male non-Asian subject weighing 77.2 kg. Inter-individual variability was estimated to be 47% for CL/F and 59% for Vc/F. The absorption and formation rate constant was 0.29 h^{-1} with an inter-individual variability of 86%.

References

1. Houk BE, Bello CL, Kang D, Amantea M (2009) A population pharmacokinetic meta-analysis of sunitinib malate (SU11248) and its primary metabolite (SU12662) in healthy volunteers and oncology patients. Clin Cancer Res 15:2497-2506. doi: 10.1158/1078-0432.ccr-08-1893

Reza Khosravan, Steven G. DuBois, Katherine Janeway, and Erjian Wang
Correspondence: R. Khosraven, MD, Pfizer Inc, Reza.Khosravan@pfizer.com

Online Resource 3 Subject baseline characteristics: categorical variables

Variable	Categories	*n* (%)
Sunitinib dosage	12.5 mg	9 (1.78)
	25 mg	37 (7.3)
	37.5 mg	26 (5.1)
	50 mg	381 (75.3)
	75 mg	23 (4.5)
	150 mg	17 (3.4)
	Other	13 (2.6)
Race	White	394 (77.9)
	Black	25 (4.9)
	Asian	57 (11.3)
	Hispanic	23 (4.5)
	Unknown	7 (1.4)
Sex	Male	308 (60.9)
	Female	198 (39.1)
ECOG PS	0	224 (44.2)
	1	264 (52.1)
	2	6 (1.2)
	Unknown	12 (2.4)
Tumor type	Solid tumor	134 (26.5)
	GIST	372 (73.5)
Treatment schedule	4/2	379 (74.9)
	2/1	56 (11.1)
	2/2	45 (8.9)
	CDD	26 (5.1)
Formulation	Intact capsule	494 (97.4)
	Sprinkled capsule	12 (2.3)

2/1 2-weeks on treatment followed by 1-week off treatment, *2/2* 2-weeks on treatment followed by 2-weeks off treatment, *4/2* 4-weeks on treatment followed by 2-weeks off treatment, *CDD* continuous daily dosing, *ECOG PS* Eastern Cooperative Oncology Group performance status

Extrapolation of Pharmacokinetics and Pharmacodynamics of Sunitinib for Children With Gastrointestinal Stromal Tumors

Cancer Chemotherapy and Pharmacology

Reza Khosravan, Steven G. DuBois, Katherine Janeway, and Erjian Wang

Correspondence: R. Khosraven, MD, Pfizer Inc, Reza.Khosravan@pfizer.com

Online Resource 4 Goodness-of-fit diagnostic plots for **(a)** plasma concentrations of sunitinib (final PK model); **(b)** plasma concentrations of the sunitinib active metabolite SU12662 (final PK model); **(c)** efficacy endpoint sum of longest diameter in target lesions; and **(d–k)** safety endpoints (**c–k**, all final PK/PD model).

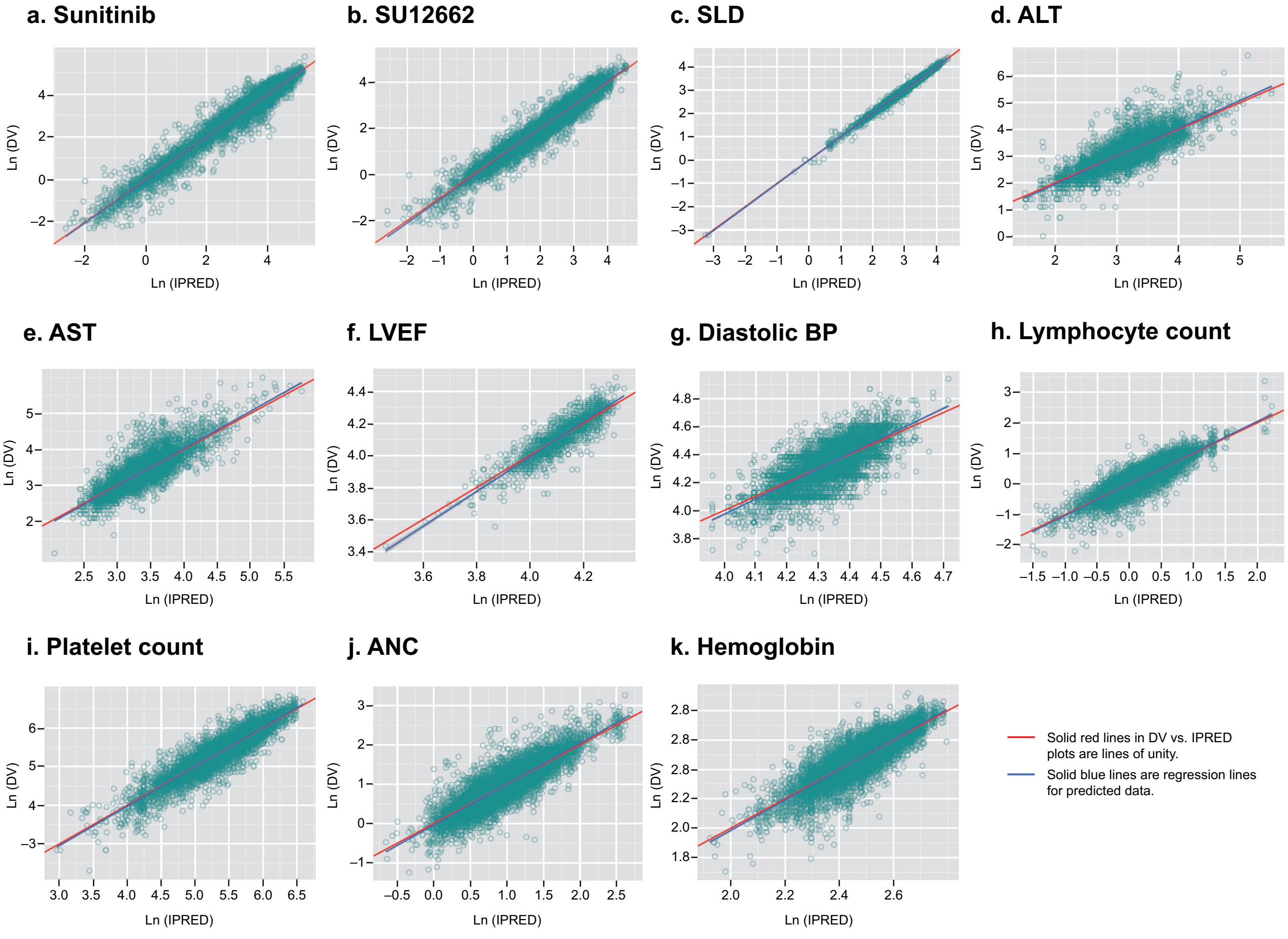

ALT alanine aminotransferase, *ANC* absolute neutrophil count, *AST* aspartate aminotransferase, *BP* blood pressure, *DV* observed, *LVEF* left-ventricular ejection fraction, *PD* pharmacodynamic, *PK* pharmacokinetic, *SLD* sum of longest diameters

Extrapolation of Pharmacokinetics and Pharmacodynamics of Sunitinib for Children With Gastrointestinal Stromal Tumors

Cancer Chemotherapy and Pharmacology

Reza Khosravan, Steven G. DuBois, Katherine Janeway, and Erjian Wang
Correspondence: R. Khosraven, MD, Pfizer Inc, Reza.Khosravan@pfizer.com

Online resource 5 Goodness-of-fit diagnostic plots observed vs. population predicted for **(a)** plasma concentrations of sunitinib (final PK model); **(b)** plasma concentrations of the sunitinib active metabolite SU12662 (final PK model); **(c)** efficacy endpoint sum of longest diameter in target lesions; and **(d–k)** safety endpoints (**c–k**, all final PK/PD model).

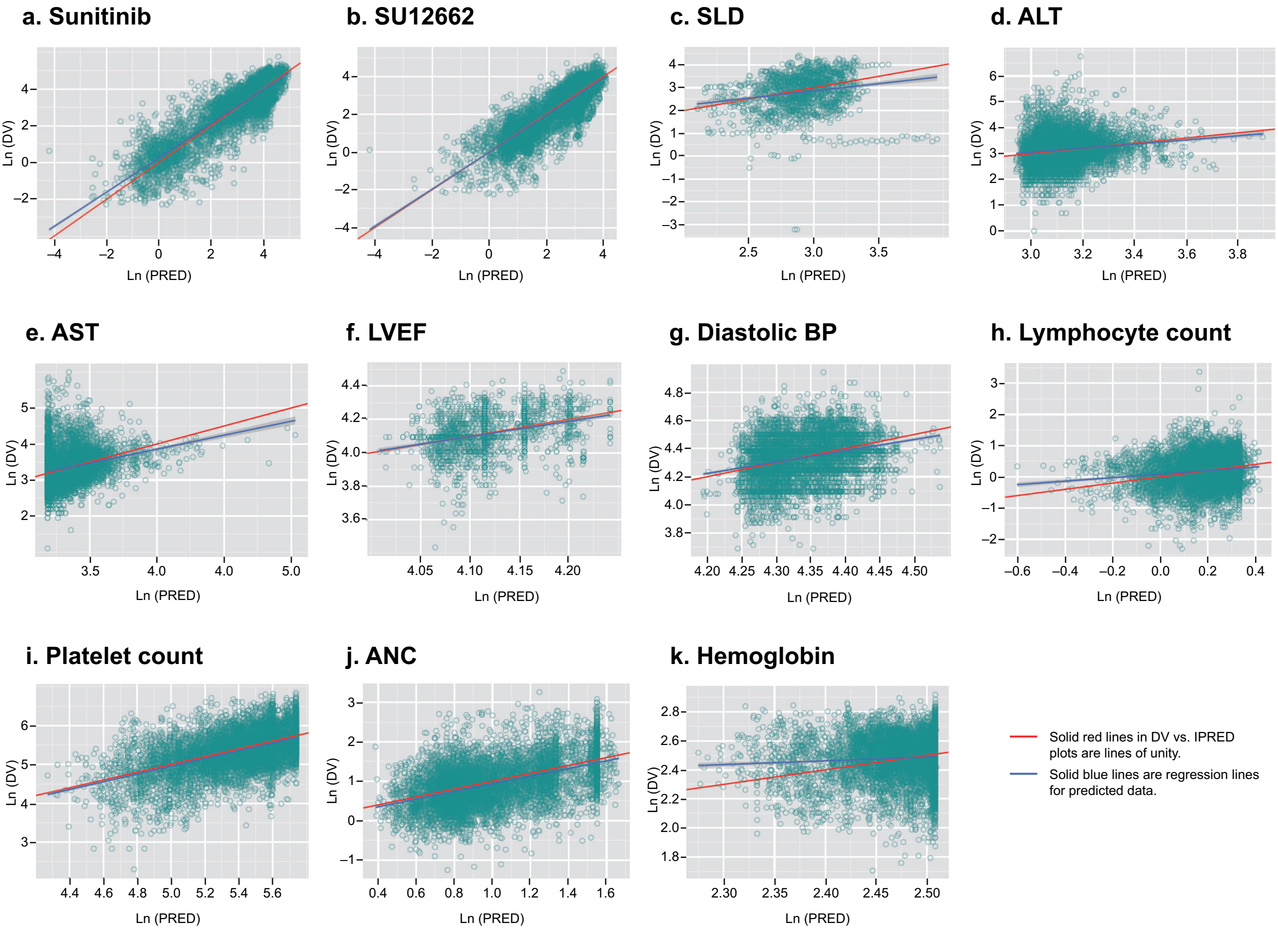

ALT alanine aminotransferase, *ANC* absolute neutrophil count, *AST* aspartate aminotransferase, *BP* blood pressure, *DV* observed, *LVEF* left-ventricular ejection fraction, *PD* pharmacodynamic, *PK* pharmacokinetic, *PRED* predicted, *SLD* sum of longest diameters

Extrapolation of Pharmacokinetics and Pharmacodynamics of Sunitinib for Children With Gastrointestinal Stromal Tumors
Cancer Chemotherapy and Pharmacology
Reza Khosravan, Steven G. DuBois, Katherine Janeway, and Erjian Wang
Correspondence: R. Khosraven, MD, Pfizer Inc, Reza.Khosravan@pfizer.com

Online Resource 6 Goodness-of-fit diagnostic plots for (**a**) plasma concentrations of sunitinib (final PK model); (**b**) plasma concentrations of the sunitinib active metabolite SU12662 (final PK model); (**c**) efficacy endpoint sum of longest diameter in target lesions and (**d**-**k**) safety endpoints (**c**-**k**, all final PK/PD model).

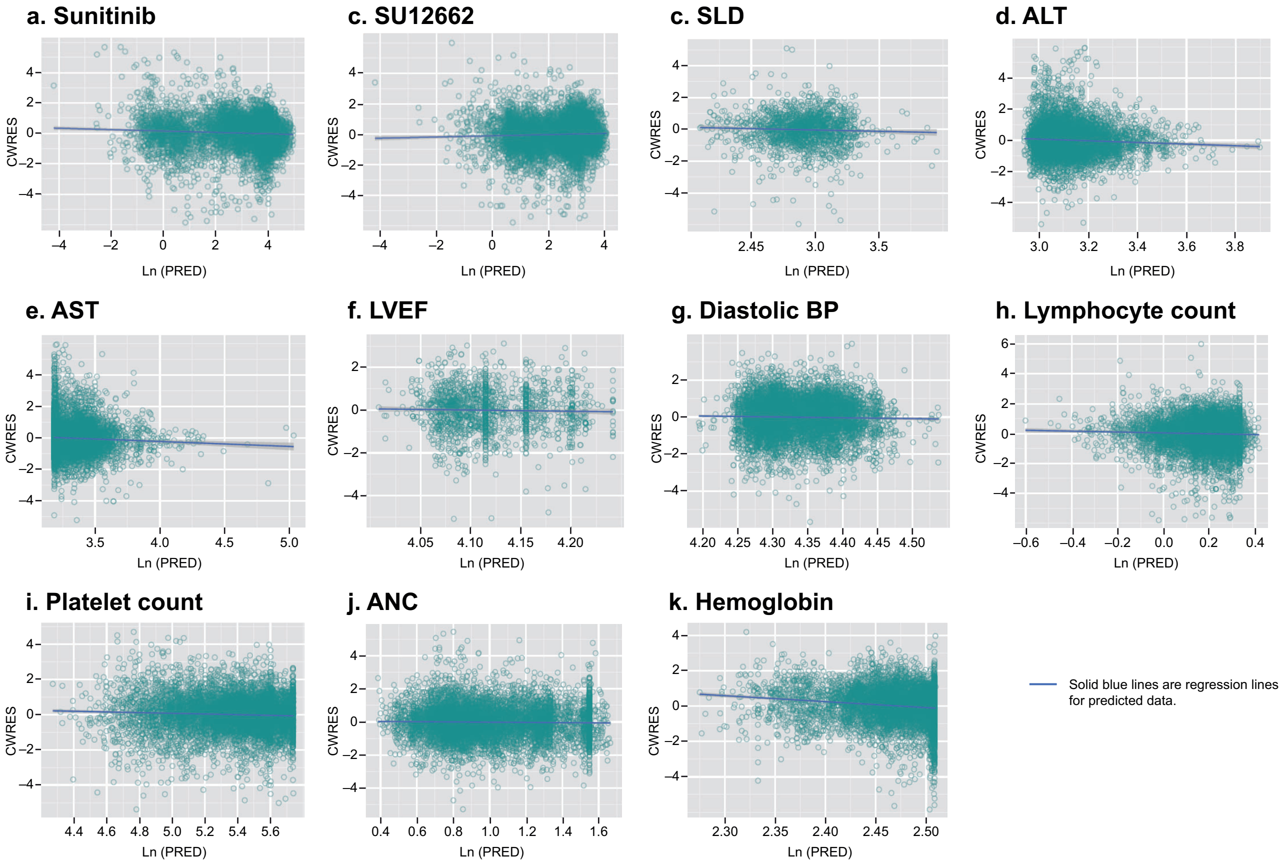

ALT alanine aminotransferase, *ANC* absolute neutrophil count, *AST* aspartate aminotransferase, *BP* blood pressure, *CWRES*, condition weighted residuals, *LVEF* left-ventricular ejection fraction, *PD* pharmacodynamic, *PK* pharmacokinetic, *PRED* predicted, *SLD* sum of longest diameters

Extrapolation of Pharmacokinetics and Pharmacodynamics of Sunitinib for Children With Gastrointestinal Stromal Tumors

Cancer Chemotherapy and Pharmacology

Reza Khosravan, Steven G. DuBois, Katherine Janeway, and Erjian Wang
Correspondence: R. Khosraven, MD, Pfizer Inc, Reza.Khosravan@pfizer.com

Online Resource 7 Goodness-of-fit diagnostic plots for (**a**) plasma concentrations of sunitinib (final PK model); (**b**) plasma concentrations of the sunitinib active metabolite SU12662 (final PK model); (**c**) efficacy endpoint sum of longest diameter in target lesions and (**d**-**k**) safety endpoints (**c**-**k,** all final PK/PD model).

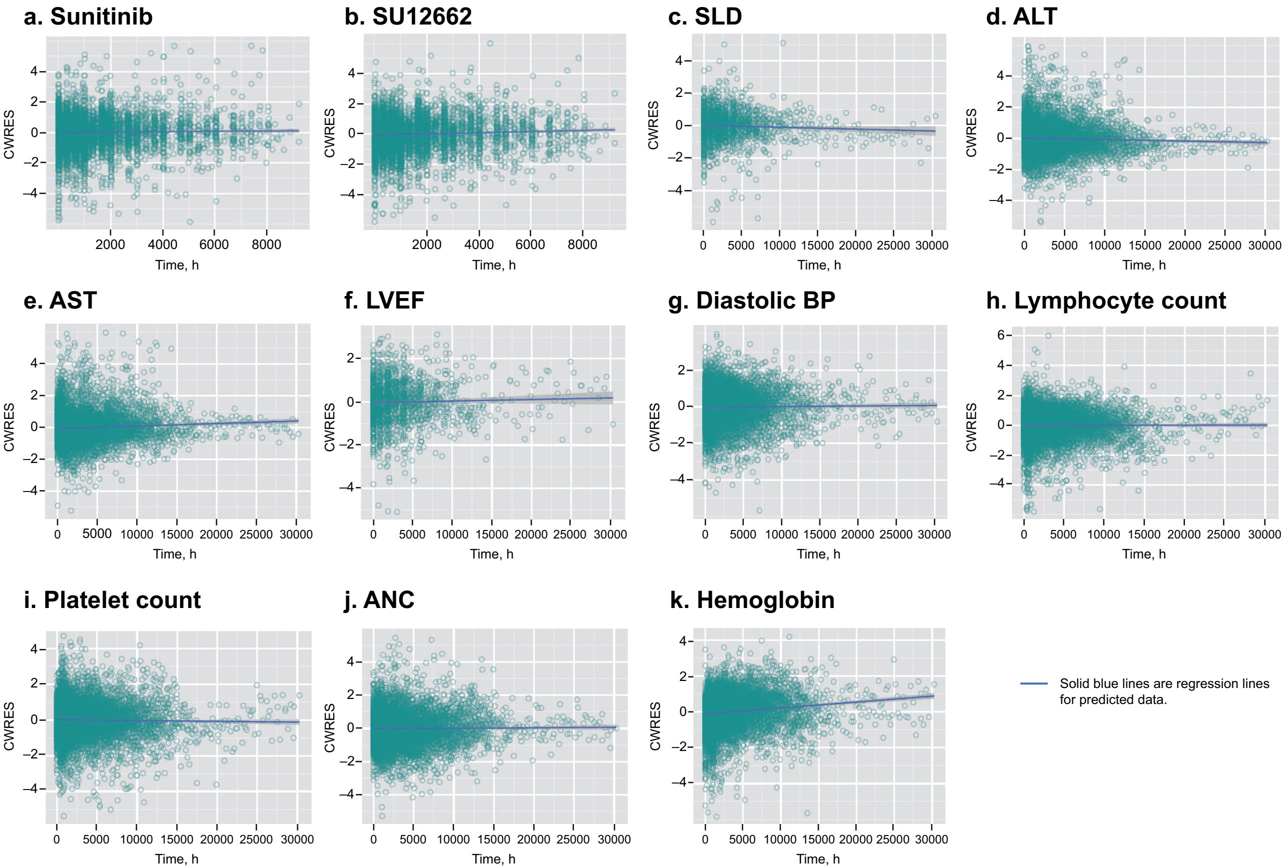

ALT alanine aminotransferase, *ANC* absolute neutrophil count, *AST* aspartate aminotransferase, *BP* blood pressure, *CWRES* condition weighted residuals, *LVEF* left-ventricular ejection fraction, *PD* pharmacodynamic, *PK* pharmacokinetic, *SLD* sum of longest diameters

Reza Khosravan, Steven G. DuBois, Katherine Janeway, and Erjian Wang
Correspondence: R. Khosraven, MD, Pfizer Inc, Reza.Khosravan@pfizer.com

Online Resource 8 Categorical safety endpoints: mean and 95% CI results for base and final models

	Population mean estimates (95% CI)			
	Base model		Final model	
**Parameter**	**Model results[a]**	**Bootstrap results[b]**	**Model results[a]**	**Bootstrap results[b]**
**HFS**				
BASE1 (θ1)	–2.8	–2.83	–2.67	–2.67
	(–3.37, –2.23)	(–3.47, –2.3)	(–3.23, –2.09)	(–3.28, –2.2)
BASE2 (θ2)	–0.75	–0.77	–0.92	–0.94
	(–0.96, –0.54)	(-0.98, –0.55)	(–1.18, –0.66)	(–1.12, –0.69)
BASE3 (θ3)	–0.84	–0.84	–1.02	–1.02
	(–1.14, –0.54)	(–1.17, –0.57)	(–1.38, –0.66)	(–1.45, –0.69)
k_{PD} (θ4)	0.038	0.039	0.025	0.026
	(0.02, 0.05)	(0.027, 0.054)	(0.01, 0.04)	(0.014, 0.04)
θRAC(k_{PD}) (θ5)	NA	NA	2.11	2.12
			(0.82, 3.4)	(1.16, 4.19)
**Fatigue**				
BASE2 (θ1)	–0.88	–0.93	–1.06	–1.14
	(–1.12, –0.64)	(–1.74, –0.65)	(–1.31, –0.81)	(–1.72, –0.83)
BASE3 (θ2)	–1.39	–1.42	–1.43	–1.45
	(–1.64, –1.14)	(–1.68, –1.17)	(–1.68, –1.14)	(–1.73, –1.18)
k_{PD} (θ3)	0.004	0.005	0.008	0.01
	(0, 0.01)	(0.002, 0.02)	(0, 0.01)	(0.002, 0.02)
θAge(k_{PD}) (θ5)	NA	NA	1.83	1.55
			(0.91, 2.75)	(–0.258, 2.61)
**Nausea**				
BASE1 (θ1)	–1.8	–1.82	–2.0	–2.04
	(–2.19, –1.41)	(–2.39, –1.41)	(–2.4, –1.6)	(–2.6, –1.62)
BASE2 (θ2)	–1.69	–1.69	–1.74	–1.74
	(–2.0, –1.38)	(–2.04, –1.42)	(–2.05, –1.43)	(–2.1, –1.49)
BASE3 (θ3)	–1.92	–1.95	–1.98	–2.03
	(–2.61, –1.23)	(–2.91, –1.39)	(–2.68, –1.28)	(–2.97, –1.45)
k_{PD} (θ4)	0.024	0.025	0.023	0.023
	(0.02, 0.03)	(0.02, 0.04)	(0.01, 0.03)	(0.02, 0.04)
θECOG(k_{PD}) (θ5)	NA	NA	0.913	0.888
			(0.38, 1.45)	(0.38, 1.63)
**Vomiting**				
BASE1 (θ1)	–2.82	–2.81	–2.82	–2.81
	(–3.32, –2.32)	(–3.46, –2.34)	(–3.32, –2.32)	(–3.46, –2.34)
BASE2 (θ2)	–1.36	–1.37	–1.36	–1.37
	(–1.67, –1.05)	(–1.72, –1.08)	(–1.67, –1.05)	(–1.72, –1.08)
BASE3 (θ3)	–2.45	–2.51	–2.45	–2.51
	(–3.42, –1.48)	(–4.05, –1.71)	(–3.42, –1.48)	(–4.05, –1.71)
k_{PD} (θ4)	0.038	0.038	0.038	0.038
	(0.03, 0.05)	(0.028, 0.05)	(0.03, 0.05)	(0.028, 0.05)

[a] 95% CI was estimated as (mean–1.96*SE – mean+1.96*SE).
[b] All bootstrap runs were successful. The numbers represent median (2.5%ile, 97.5%ile).
BASE baseline, *CI* confidence interval, *HFS* hand–foot syndrome, *ECOG* Eastern Cooperative Oncology Group performance status, k_{PD} first-order rate constant, *NA* not applicable, *RAC* race, *SE* standard error

Reza Khosravan, Steven G. DuBois, Katherine Janeway, and Erjian Wang
Correspondence: R. Khosraven, MD, Pfizer Inc, Reza.Khosravan@pfizer.com

Online Resource 9 Pediatric GIST Patient Characteristics from Janeway et al. and Agaram et al.

		Sunitinib dosage					
Age at study entry, y	Sex	Response to sunitinib	TTP, mo	Starting dose, mg/d	Maximum dose, mg/d	Starting dose, mg/m^2	Maximum dose, mg/m^2
**Janeway et al. [1]**							
17	F	SD	7	37.5	50	21	28
10	F	PR[a]	>21	25	37.5	20	30
16	F	SD	8	25	25	18	18
16	M	SD	18	50	50	33	33
16	F	SD	>18	50	50	32	32
16	M	PD	<1	37.5	37.5	NA	NA
14	F	SD	18	25	50	21	41
**Agaram et al. [2]**							
10	F	SD	8	25	NA	NA	NA
16	F	intolerant	1	37.5	NA	NA	NA
14	F	PD	5	25	NA	NA	NA
18	F	PR	8	50	NA	NA	NA

[a] Overall SD with complete lung met response.
F female, *GIST* gastrointestinal stromal tumor, *M* male, *NA* not available, *PD* progressive disease, *PR* partial response, *SD* stable disease, *TTP* time to progression

References

1. Janeway KA, Albritton KH, Van Den Abbeele AD, D'Amato GZ, Pedrazzoli P, Siena S, Picus J, Butrynski JE, Schlemmer M, Heinrich MC et al (2009) Sunitinib treatment in pediatric patients with advanced GIST following failure of imatinib. Pediatr Blood Cancer 52:767-771. doi: 10.1002/pbc.21909
2. Agaram NP, Laquaglia MP, Ustun B, Guo T, Wong GC, Socci ND, Maki RG, DeMatteo RP, Besmer P, Antonescu CR (2008) Molecular characterization of pediatric gastrointestinal stromal tumors. Clin Cancer Res 14:3204-3215. doi: 10.1158/1078-0432.ccr-07-1984

Reza Khosravan, Steven G. DuBois, Katherine Janeway, and Erjian Wang
Correspondence: R. Khosraven, MD, Pfizer Inc, Reza.Khosravan@pfizer.com

Online Resource 10 The predicted median for PK/safety/efficacy following multiple dosing with sunitinib in children reported in Janeway et al. [1] and Agaram et al. [2] studies and 50 mg/day in adults on Schedule 4/2

PK/PD endpoint	Median at baseline All ages	Median (95% CI) for each PK/PD endpoint at cycle 6 day 27/28	
		Janeway/Agaram	Adults
ALT, U/L	20.5	23.3 (20.9, 74.3)	23.8 (20.9, 109)
ANC, 109/L	4.71	2.36 (1.49, 3.69)	2.32 (1.55, 3.53)
AST, U/L	24.2	29.7 (25.3, 119)	30.8 (25.5, 127)
BP, mmHg	72.8	79.5 (74.9, 92.1)	80.6 (75.3, 95.0)
Hemoglobin, g/dL	12.3	11.8 (7.14, 12.3)	11.7 (6.62, 12.3)
LVEF, %	61.2	58.9 (42.9, 60.9)	58.5 (42.7, 60.8)
Lymphocyte count, 109/L	1.39	1.25 (0.832, 1.36)	1.24 (0.817, 1.35)
Platelet count, 109/L	311	216 (112, 283)	165 (51, 302)
SLD, % change from baseline	0.00	0.69 (–64.8, 91.0)	0.083 (–62.4, 96.3)
Sunitinib trough concentration, ng/mL	0.00	38.5 (13.5, 93.7)	44.7 (17.9, 106)
Sunitinib average concentration, ng/mL	0.00	43.3 (18.3, 99.8)	49.0 (22.7, 110)
SU012662 trough concentration, ng/mL	0.00	18.7 (6.04, 52.0)	19.7 (6.78, 52.6)
SU012662 average concentration, ng/mL	0.00	19.8 (7.13, 54.1)	20.9 (7.72, 54.2)

This table shows the predicted median (95% CI) for PK/safety/efficacy on day 28 of cycle 6 based on the pooled data from all trial simulations following multiple dosing with sunitinib starting doses in Janeway et al. [1] and Agaram et al. [2] in children and 50 mg/day in adults on Schedule 4/2 (4 weeks on followed by 2 weeks off). Baseline was set to the final model population baseline mean value for comparison of predicated relative changes of each endpoint across different age groups; median (95% CI) represents median (2.5%ile, 97.5%ile); sunitinib average concentration median (95% CI) represents mean of median (2.5%ile, 97.5%ile) values at 0, 3, 6, 9, 12, and 24 hours post dose on day 27 of cycle 6.
ALT alanine amino transferase, *ANC* absolute neutrophil count, *AST* aspartate aminotransferase, *BP* diastolic blood pressure, *CI* confidence interval, *LVEF* left-ventricular ejection fraction, *PD* pharmacodynamics, *PK* pharmacokinetics, *SLD* single longest diameter

References

1. Janeway KA, Albritton KH, Van Den Abbeele AD, D'Amato GZ, Pedrazzoli P, Siena S, Picus J, Butrynski JE, Schlemmer M, Heinrich MC et al (2009) Sunitinib treatment in pediatric patients with advanced GIST following failure of imatinib. Pediatr Blood Cancer 52:767-771. doi: 10.1002/pbc.21909
2. Agaram NP, Laquaglia MP, Ustun B, Guo T, Wong GC, Socci ND, Maki RG, DeMatteo RP, Besmer P, Antonescu CR (2008) Molecular characterization of pediatric gastrointestinal stromal tumors. Clin Cancer Res 14:3204-3215. doi: 10.1158/1078-0432.ccr-07-1984
